# Hereditary Angioedema Caused By C1-Esterase Inhibitor Deficiency: A Literature-Based Analysis and Clinical Commentary on Prophylaxis Treatment Strategies

**DOI:** 10.1097/1939-4551-4-S2-S9

**Published:** 2011-02-15

**Authors:** Richard G Gower, Paula J Busse, Emel Aygören-Pürsün, Amin J Barakat, Teresa Caballero, Mark Davis-Lorton, Henriette Farkas, David S Hurewitz, Joshua S Jacobs, Douglas T Johnston, William Lumry, Marcus Maurer

**Affiliations:** 1 Clinical Associate Professor of Medicine, University of Washington, Marycliff Allergy Specialists, PS, Spokane, WA; 2 Assistant Professor, Department of Medicine - Allergy & Immunology, The Mount Sinai Medical Center, New York, NY; 3 Klinikum der Johann Wolfgang Goethe-Universität, Frankfurt, Germany; 4 Clinical Professor of Pediatrics, Georgetown University Medical Center, Washington, DC; 5 Hospital La Paz Health Research Institute (IdiPAZ), Allergy Service, Madrid, Spain; 6 Winthrop Rheumatology, Allergy and Immunology, Mineola, NY; 7 Associate Professor of 3rd Department of Internal Medicine, Semmelweis University, Budapest, Hungary; 8 Clinical Professor of Medicine, Allergy Clinic of Tulsa, Tulsa, OK; 9 Allergy & Asthma Medical Group, Walnut Creek, CA; 10 Allergy Partners, PA, Greenville, SC; 11 Clinical Professor Internal Medicine/Allergy Division, University of Texas Southwestern Medical School, Asthma and Allergy Research Associates, Dallas, TX; 12 Professor of Dermatology and Allergy, Allergie-Centrum-Charité/ECARF, Charité-Universitätsmedizin Berlin, Berlin, Germany

**Keywords:** C1-esterase inhibitor, attenuated androgen, angioedema, HAE, prophylaxis

## Abstract

Hereditary angioedema (HAE) caused by C1-esterase inhibitor deficiency is an autosomal-dominant disease resulting from a mutation in the C1-inhibitor gene. HAE is characterized by recurrent attacks of intense, massive, localized subcutaneous edema involving the extremities, genitalia, face, or trunk, or submucosal edema of upper airway or bowels. These symptoms may be disabling, have a dramatic impact on quality of life, and can be life-threatening when affecting the upper airways. Because the manifestations and severity of HAE are highly variable and unpredictable, patients need individualized care to reduce the burden of HAE on daily life. Although effective therapy for the treatment of HAE attacks has been available in many countries for more than 30 years, until recently, there were no agents approved in the United States to treat HAE acutely. Therefore, prophylactic therapy is an integral part of HAE treatment in the United States and for selected patients worldwide. Routine long-term prophylaxis with either attenuated androgens or C1-esterase inhibitor has been shown to reduce the frequency and severity of HAE attacks. Therapy with attenuated androgens, a mainstay of treatment in the past, has been marked by concern about potential adverse effects. C1-esterase inhibitor works directly on the complement and contact plasma cascades to reduce bradykinin release, which is the primary pathologic mechanism in HAE. Different approaches to long-term prophylactic therapy can be used to successfully manage HAE when tailored to meet the needs of the individual patient.

## 

**H**ereditary angioedema (HAE) caused by C1-esterase inhibitor deficiency is an autosomal-dominant disease resulting from a mutation in the C1-inhibitor gene[1,2]. Although HAE is an inherited disorder, 25% of cases arise from spontaneous mutations[3]. HAE is characterized by recurrent attacks of intense, massive, localized subcutaneous edema, without pruritus, involving the extremities, genitalia, face, or trunk, or submucosal edema of the upper airway or bowels[2,4]. There is a scarcity of epidemiologic studies on HAE prevalence. Studies based on national HAE registries show a minimal prevalence ranging from 1.09 to 1.51 in 100,000 inhabitants,[5-7] with the actual prevalence expected to be higher. Estimates indicate that approximately 1 in 50,000 people in the general population has HAE[8]. No sex or race predominance has been described[3].

Mutations in the C1-inhibitor gene located on chromosome 11 cause 2 major forms of HAE: type I and type II[9]. In type I HAE, which accounts for approximately 85% of HAE cases, low C1-esterase inhibitor levels result from a deficiency in the amount of C1-esterase inhibitor produced. Both functional and antigenic C1-esterase inhibitor levels are reduced[10,11]. Type II HAE accounts for 15% of cases and is characterized by normal or elevated antigenic C1-esterase inhibitor with low levels of functional C1-esterase inhibitor[2,12,13]. The 2 types of HAE are alike in clinical presentation, but are caused by different mutations. According to the C1-inhibitor gene mutation database, more than 275 different mutations have been identified[14]. Most mutations are small deletions, insertions, or point mutations, however, larger rearrangements of the gene with partial duplications or deletions account for 15 to 20% of all mutations leading to HAE[13,15-17].

A rare third type of HAE does not exhibit a deficiency in C1-esterase inhibitor[18,19]. HAE with normal C1-esterase inhibitor may be associated with mutations in the coagulation factor XII gene but there are patients who do not exhibit any genetic mutations[18,20]. This communication restricts the discussion to the type I and type II HAE caused by deficiency of functional C1-esterase inhibitor.

To review the current status of prophylactic management of HAE, an international panel of experts was assembled in Philadelphia, PA, on August 13-14, 2010. Because of different approaches to management of HAE in various countries, these proceedings attempt to reflect the spectrum of prophylaxis treatment options with a focus on androgen and C1-esterase inhibitor therapy.

## Clinical Presentation

**Figure 1:**
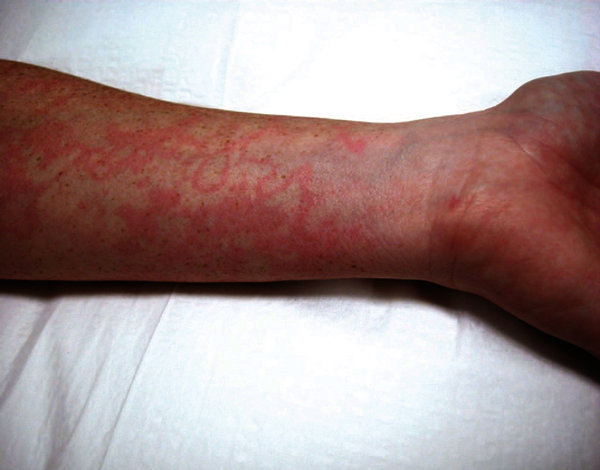
**Erythema marginatum on the arm of a patient with hereditary angioedema**. Note that lesions are neither raised nor pruritic. Photography courtesy of William R. Lumry, MD.

The symptoms of both type I and type II HAE are indistinguishable. Although the initial symptoms of HAE can occur at any age, symptoms usually first appear in childhood, worsen during puberty, and persist throughout life, with attack frequency and severity varying from patient to patient. HAE is not generally diagnosed at initial presentation, and the time of diagnosis has been shown to range from 8 to 22 years from the first attack[6,21,22]. Attacks often occur without a trigger; however, precipitating factors that have been shown to contribute to the frequency of attacks include stress, trauma, infection, menstruation, and pregnancy[3,4,9,21,23]. Various medications, such as estrogen-containing agents and angiotensin-converting enzyme inhibitors, may also induce HAE attacks[3,4,9,21,23]. Before attacks, many patients experience prodromal symptoms that can include tingling sensations or erythema marginatum, a nonpruritic and not raised rash (Figure 1)[4,21,24]. The patient's family history or frequency and severity of attacks do not predict the course of future attacks nor do they predict involvement of the airway during an attack[25]. 

The cardinal symptoms of HAE include episodic, localized, nonurticarial, nonpitting subcutaneous edema of the skin (hands, arms, legs, feet, trunk, face, genitalia) and submucosal edema of the bowels and upper airway[2-4,9]. Attacks may affect several sites of the body simultaneously or consecutively. Edema typically progresses slowly, peaks over the first 24 to 36 hours, and usually resolves within 72 hours, but can persist for as long as 1 week. HAE attacks can be painful and disfiguring but are usually not life-threatening. Attacks affecting the upper airways, however, can lead to obstruction and suffocation, and the manifestations of gastrointestinal edema (ie, abdominal attacks) can include intractable abdominal pain, vomiting, nausea, diarrhea, and intestinal obstruction, and potentially can lead to hypovolemic shock[2-4,9,26,27].

**Figure 2:**
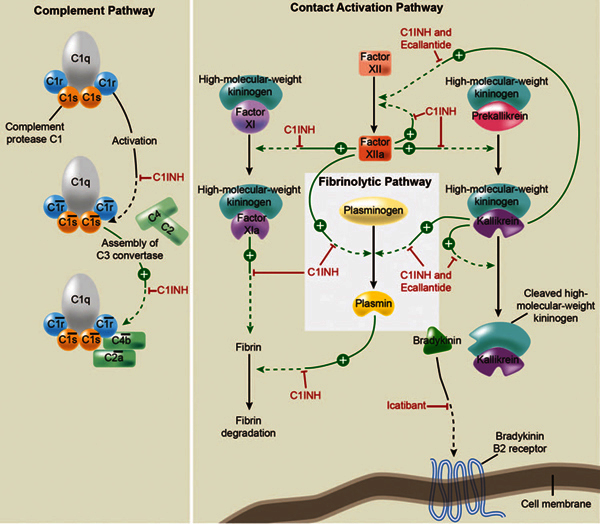
**Pathways inhibited by C1-esterase inhibitor (C1INH) (reproduced from Zuraw,**[2]**with permission)**.

During an attack, the activation of the complement and contact cascades and the inadequate response by C1-esterase inhibitor cause an overproduction of bradykinin (Figure 2)[2]. Increased bradykinin levels increase vascular permeability and extravasation, manifesting as edema (Figure 2)[1,28-30]. This is an important differentiating feature of HAE when compared with allergic reactions, which are primarily mediated by histamine. During an allergic reaction, IgE antibodies react with specific allergens, inducing histamine release from mast cells[9]. This reaction leads to angioedema and/or urticaria, which subside with use of epinephrine, antihistamines, or corticosteroids. In contrast, HAE manifestations do not respond to such therapies because symptoms and swelling are mediated by bradykinin[9].

## Consequences

The consequences of HAE are considerable. HAE may account for 15,000 to 30,000 emergency department visits annually in the United States alone[31,32]. Laryngeal edema presents the greatest risk to patients, and approximately 50% of patients with HAE have at least 1 laryngeal attack in their lifetime[9]. In the past, fatality from asphyxiation during a laryngeal attack was reported in approximately 30% of patients with HAE[9]. Fatal laryngeal attacks still may occur, particularly in the absence of a proper diagnosis or in patients who do not receive appropriate or timely treatment[2,33]. Patients with HAE are often misdiagnosed, resulting in unnecessary medical and surgical interventions[22,31,34]. Abdominal symptoms may mimic an acute appendicitis or other forms of acute abdomen and lead to unnecessary abdominal surgery[2,8,22]. According to 1 estimate, 45% of patients presenting to an emergency department with an HAE attack are subsequently hospitalized[35].

The symptoms of HAE may be disabling and have a dramatic impact on quality of life[36]. Swelling in the hands and feet can be debilitating, abdominal attacks often cause extreme pain, and facial attacks are generally disfiguring and may extend to involve the larynx[21]. HAE attacks typically incapacitate patients for 20 to 100 days annually depending on attack severity, frequency, and duration[13]. HAE significantly impacts the ability of a patient to work or go to school; these impairments are comparable to those experienced by patients with severe asthma or Crohn's disease[36]. Patients may develop narcotic dependence while trying to appropriately manage frequent and severe abdominal pain[37]. Additional care may be required to manage the psychologic impact of HAE disease[37]. Patients with HAE are more likely to suffer symptoms of depression than the general population, with 42.5% of patients showing at least mild symptoms of depression[36]. Data suggest that patients with more severe disease are more likely to experience depression than those with mild or infrequent attacks[36]. Patients with HAE are nearly twice as likely to report taking psychotropic or antidepressant medication compared with the general population[36].

## Diagnosis

Identifying HAE may be difficult because of variability in clinical presentation. HAE should be suspected in patients presenting with any of the characteristic symptoms, especially in the presence of a positive family history[25]. Screening for genetic mutations is not generally performed for diagnostic reasons. Instead, laboratory testing is indicated for patients with suspected HAE. Biochemical markers used for the diagnosis of HAE are serum complement factor 4 (C4), C1-esterase inhibitor antigen, functional C1-esterase inhibitor, and C1q antigenic protein[25]. The most cost-effective method to screen for HAE is measurement of C4 levels[2]. If C4 is normal, as can occur in some patients between attacks, the measurement should be repeated during an acute attack[25]. Whether to obtain C1-esterase inhibitor antigen and functional C1-esterase inhibitor studies at the time of C4 collection or at a subsequent time can be based on the index of suspicion. Low levels of C4, C1-esterase inhibitor antigenic protein, and functional C1-esterase inhibitor are consistent with a type I HAE diagnosis but should be confirmed via a second measurement. Low levels of C4 and functional C1-esterase inhibitor with normal or elevated C1-esterase inhibitor antigenic protein are indicative of type II HAE[25]. C1q antigenic protein is normal in HAE[25]. It should be noted that variability in symptoms is not related to the levels of biochemical markers and that patients with lower levels of the markers outlined here do not necessarily exhibit more severe HAE symptoms than those with higher levels[2]. A recent study, however, found a significant correlation between severity scores and baseline functional C1-esterase inhibitor levels, which suggests the potential significance of monitoring functional C1-esterase levels in relation to clinical disease course[38].

## Treatment Approaches

The goals of treatment for HAE are focused on life-saving efforts, slowing the progression and severity of attacks, and reducing the number of attacks and their impact on patient quality of life. Because of large variations in clinical presentation and severity of disease, HAE treatment is individualized and based on a close collaboration between physician and patient.

The pharmacotherapy of HAE can be categorized into 3 approaches: acute treatment, short-term prophylaxis, and long-term prophylaxis. Acute treatment options for HAE include C1-esterase inhibitor (human or recombinant) replacement therapy, icatibant, or ecallantide (Table 1)[2,9,25,39,48]; however, it should be noted that not all agents are currently approved in all countries. Infusions of C1-esterase inhibitor concentrate have been shown to increase functional levels of C1-esterase inhibitor and C4 to near-normal levels[49]. Icatibant is a bradykinin B2-receptor antagonist that reverses increased vascular permeability and inhibits vasodilation and extravasation[40]. Ecallantide is a human plasma kallikrein inhibitor that treats HAE symptoms by directly inhibiting plasma kallikrein and decreasing the conversion of high-molecular-weight kininogen to bradykinin[41]. Fresh frozen plasma has been used, but its utility is limited because it contains additional kinins and complement factors, posing a potential threat of worsening HAE symptoms[3,34]. Nevertheless, successful use of fresh frozen plasma has been reported for both acute treatment and prophylaxis[50-52]. HAE is mediated by bradykinin, therefore, it is noteworthy to mention that treatments used for other forms of angioedema, including antihistamines, epinephrine, and corticosteroids, are ineffective in treating HAE-related angioedema and should be avoided[4].

**Table 1:** Drugs Commonly Used for Acute and Prophylactic Treatment of HAE[2,9,25,45-48]

Drug Class or Name	Adult Dosage and Route of Administration	Mechanism of Action
Acute therapy		
C1-esterase inhibitor (human)	20 U/kg IV	Replaces missing or malfunctioning C1-esterase inhibitor
Ecallantide	30 mg SC split into 3 injections	Potent, selective, reversible inhibitor of plasma kallikrein, which reduces the conversion of high-molecular-weight kininogen to bradykinin
Icatibant	30 mg SC	Selective competitive bradykinin type 2 receptor antagonist
Prophylactic therapy		
17-alpha alkylated androgens		Exact mechanism not known. Thought to increase endogenous C1-esterase inhibitor levels via hepatic synthesis and a subsequent increase in the expression of mRNA
Danazol	100 mg PO every 3 days to 600 mg QD	
Oxandrolone	2.5 mg PO every 3 days to 20 mg QD	
Stanozolol	1 mg PO every 3 days to 6 mg QD	
Antifibrinolytics		Inhibit the formation and activity of plasmin and subsequently decrease plasmin-induced activation of C1
Tranexamic acid	20-50 mg/kg/d PO split BID or TID	
*ε*-aminocaproic acid	8 to 12 g PO daily in 4 divided doses	
Nanofiltered C1-esterase inhibitor (human)	1000 U IV every 3 to 4 days	Replaces missing or malfunctioning C1-esterase inhibitor

BID, twice daily; HAE, hereditary angioedema; IV, intravenous; PO, by mouth; QD, once daily; SC, subcutaneous; TID, 3 times daily; U, units.

The goal of short-term, or procedural, prophylaxis is to prevent an HAE attack in patients that may be triggered by medical, surgical, or dental procedures[4,53]. C1-esterase inhibitor, attenuated androgens, antifibrinolytics, icatibant, and fresh frozen plasma have been used successfully for short-term prophylaxis[2,8,25,54]. Consensus guidelines recommend that patients with HAE receive prophylactic treatment with 500 to 1,500 U of C1-esterase inhibitor 1 to 6 hours before the procedure[25]. If C1-esterase inhibitor is not available, the guidelines recommend treatment with an attenuated androgen for 5 days before the procedure and 2 to 5 days after, or administration of fresh frozen plasma 1 to 6 hours before the procedure[25].

Although effective therapy for the treatment of HAE attacks has been available in many countries for more than 30 years, until recently, there were no agents approved in the United States to treat HAE acutely. Therefore, prophylactic therapy is an integral part of HAE treatment in the United States and for selected patients worldwide. Long-term, or routine, prophylaxis is an important treatment option for patients with HAE. Patients who can be considered for prophylactic therapy are those who experience frequent or severe attacks, have had a previous laryngeal attack, have significant anxiety and poor quality of life as a result of HAE, have limited access to emergency medical care, or choose to receive prophylaxis[37]. In addition, long-term prophylaxis is indicated in patients for whom acute therapy is ineffective or unavailable[37]. Attenuated (17-alpha alkylated) androgens have long been the gold standard for prophylaxis in numerous countries, with danazol, stanazolol, and oxandrolone being used more frequently than other androgens. Although the precise mechanism of action is not known, attenuated androgens are thought to increase endogenous C1esterase inhibitor levels via hepatic synthesis and a subsequent increase in the expression of mRNA[55,56].

Another long-term prophylaxis treatment option, recently approved for use in the United States, is nanofiltered human C1-esterase inhibitor concentrate[43]. The antifibrinolytic agents, *ε*-aminocaproic acid and tranexamic acid, are not approved in the United States but have been used extensively in some European countries for long-term prophylaxis, especially in children and pregnant women in whom androgen therapy is contraindicated because of significant risk of adverse effects[4,8,37]. Antifibrinolytics may also be useful in other patients in whom attenuated androgens or intravenous therapy is not appropriate. However, because of relatively low efficacy and poor safety profile, including the potential to cause hypotension, cardiac arrhythmias, rhabdomyolysis, and thromboembolism, the use of antifibrinolytics is limited[2,9,41,57].

Attenuated androgens and C1-esterase inhibitor are at the forefront of long-term prophylaxis therapy options for HAE, and therefore the goal of this paper is to provide an overview of the literature and clinical experience with these agents and identify patients who are candidates for each type of treatment. A discussion of other therapies is beyond the scope of this paper.

## Experience With Attenuated Androgen Therapy in the Long-Term Prophylaxis of Hae

**Figure 3:**
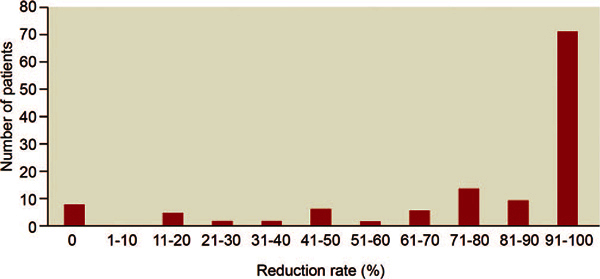
**Reduction rate of HAE attacks during long-term treatment with danazol in 118 patients (reproduced from Bork et al,**[59]**with permission)**.

Attenuated androgen therapy is the most commonly prescribed prophylactic therapy and most extensively studied, largely because until recently, it was the only approved prophylaxis treatment option for HAE in the United States[58]. Danazol, stanozolol, and oxandrolone are the attenuated androgens typically used as prophylaxis for HAE[37]. Data demonstrate that approximately 94 to 100% of patients respond to prophylactic therapy with danazol and report a decrease in frequency and severity of attacks; however, 5 to 8% of patients do not respond to danazol therapy (Figure 3)[59-62]. Although danazol is effective in reducing the number of attacks, the majority of patients treated with this agent are not symptom-free. In a large retrospective study, 24% of patients were symptom-free while taking danazol, whereas approximately 14% who responded to danazol therapy had 11 or more attacks per year[59]. Recent data suggest that long-term use of this agent may result in reduced efficacy over time. Reports indicate that in some patients the effect of danazol declines after 4 to 6 years of treatment[59,63-65]. Although attenuated androgens are not approved therapy for HAE in children, danazol has been shown to be effective prophylactic therapy in prepubescent children with severe recurrent HAE attacks[64,66]. Stanozolol and oxandrolone are preferred by some clinicians for use in children partly because of their improved safety profiles[2,67,68]. Therapy in children is complicated by the potential for androgens to affect growth and development, particularly premature closure of epiphyseal plates resulting in decreased growth[2,64]. In all patients, attenuated androgen dosing should be individualized and based on clinical response[2,25]. Furthermore, caution should be exercised when switching agents because no dose-equivalency data currently exist.

Attenuated androgens pose a significant risk for adverse events, leading some patients to refuse therapy. Side effects may include weight gain, acne, virilization, altered libido, menstrual irregularities, headaches, depression, fatigue, lipid abnormalities, hypertension, cholestasis, increased liver enzymes, peliosis hepatitis, and hepatocellular adenomas[2,8,59,69-73]. To review the published safety data on these agents, we performed a literature search and identified articles that included an evaluation of the safety of long-term attenuated androgen therapy in HAE (Table 2)[59-62,70,72-76]. We found that many patients experience adverse effects while taking attenuated androgens[59,62,72,77,78]. These adverse effects can be controlled or minimized by using the lowest effective dose, and some patients are capable of tolerating the adverse effects associated with attenuated androgen therapy. It should be noted, however, that many of the patients in these reports were treated with attenuated androgens at a time when there were very limited options for prophylactic therapy for HAE.

**Table 2:** Common Adverse Events Associated With Attenuated Androgen Therapy*

Adverse Event	Prevalence Rate (%)	Reference(s)
Weight gain	14.3-60.0	[59], [60], [62], [70], [72], [74], [75]
Acne	4.8-22.0	[59], [62], [70], [72], [75], [76]
Virilization†	1.8-46.6	[59], [60], [62], [72], [75]
Menstrual irregularities	14.4-80.0	[59-62], [70], [72], [75], [76]
Headache/migraine	13.6-49.5	[59], [62], [74], [75]
Psychological abnormalities‡	9.4-16.0	[59], [62], [72]
Arterial hypertension	25.0-30.0	[70], [74]
Lipid abnormalities	27	[73], [75]
Hepatic disease/adenomas§	1.8-40.0	[59], [60], [62], [75]
Hematuria	13	[62]

*Includes data for danazol and stanozolol. Patients may have experienced more than 1 adverse event.

†Includes voice changes, increased hair growth, decrease in breast size, clitoral hypertrophy, increase in muscle mass, laryngeal prominence.

‡Includes depression, aggressiveness, fatigue, panic attacks, mood changes.

§Includes increase in pathological laboratory findings, including hepatic enzymes.

Patient compliance with attenuated androgen therapy may be affected by considerable weight gain, the amount of which has been reported to be as high as 45 kg in 1 patient after receiving 1 year of therapy with danazol 800 mg daily[59]. Affecting women in particular, virilization symptoms such as voice changes, hirsutism, decrease in breast size, clitoral hypertrophy, increase in muscle mass, disturbances in libido, and laryngeal prominence have been reported with varying ranges of frequency and severity[58,59,72,74]. Although some studies demonstrate a prevalence rate of approximately 2%,[60] symptoms of virilization have been reported to occur in as many as 1 in 3 patients[72] and in up to 79% of female patients receiving attenuated androgens[74]. Acne resulting from attenuated androgen therapy also can be a significant source of anxiety among patients. Because of the risk of causing teratogenic effects in an unborn fetus, in particular masculinization, nonhormonal contraception or progestin-only birth control pills are recommended in fertile female patients taking attenuated androgens[79]. Many female patients also report menstrual irregularities such as menometrorrhagia or amenorrhea while receiving attenuated androgen therapy[60,75]. In male patients, loss of libido and gynecomastia have been known to occur with androgen use[58].

Other undesirable effects of androgens include headache, migraine, and alopecia[59,62,74,75]. Psychologic abnormalities such as anxiety, depression, aggressiveness, fatigue, panic attacks, and mood changes have been reported to occur in as many as 16% of patients receiving attenuated androgen therapy[59,62].

Additional serious adverse effects such as hypertension, lipid abnormalities, cystitis, hematuria, and liver disease, including adenomas and carcinoma, have also been associated with attenuated androgen therapy[59,60,62,70,73-75,80,81]. In 1 study, patients who received long-term prophylaxis for HAE with danazol were at a higher risk for developing abnormally low levels of high-density lipoprotein and high levels of low-density lipoprotein when compared with patients not taking attenuated androgens and healthy control subjects[73]. Liver disease, ranging from increased transaminases to adenomas and peliolosis hepatitis, has been reported with androgen use and warrants vigilant monitoring of liver function[59,60,62,75,82]. According to the International Consensus Algorithm for the Diagnosis, Therapy, and Management of Hereditary Angioedema,[25] patients receiving attenuated androgens should be monitored every 6 months for changes in liver function (ie, alanine aminotransferase [ALT]/ aspartate aminotransferase [AST], alkaline phosphatase), lipid profile, complete blood cell count, and urinalysis. Patients receiving more than 200 mg of danazol daily and patients who are of prepubescent age should undergo an ultrasound of the liver and spleen every 6 months and a yearly alpha fetoprotein level. Patients receiving less than 200 mg of danazol daily should undergo an ultrasound of the liver and spleen on a yearly basis[25,83].

The side effects associated with attenuated androgens may lead patients to discontinue therapy. In a large series of patients with HAE who were treated with prophylactic danazol from 2 months to 30 years, more than 25% discontinued danazol because of adverse effects and almost 10% discontinued because of a fear of adverse effects[59]. One half of patients who discontinued danazol as a result of adverse effects did so within the first 2 years of therapy. Also, the average dose of danazol was higher in patients who discontinued because of adverse effects than in patients who reported side effects but continued therapy (254 mg/d vs 154 mg/d, respectively). The side effects associated with attenuated androgens seem to be dose dependent and increase with duration of therapy[58]. This underscores the importance of titrating to the lowest dose that confers adequate prophylaxis to avoid or at least mitigate adverse effects[2,78].

## Experience with C1-Esterase Inhibitor in the Long-Term Prophylaxis of Hae

The first large-scale attempt to purify C1-esterase inhibitor was documented in 1974[84]. The development of improved manufacturing processes resulting in purified human plasma-derived C1-esterase inhibitor formulations has enabled their use for the treatment of HAE attacks since the 1980s[85]. C1-esterase inhibitor is also highly effective in patients requiring short-term prophylaxis[85-88] and its utility has been confirmed in patients with frequent attacks who experience intolerance to or lack of efficacy with danazol therapy[89,90]. However, experience with C1-esterase inhibitor in long-term prophylactic therapy is not as extensive as for acute treatment or short-term prophylaxis; long-term prophylaxis was not explored until 1989 and efficacy in a controlled trial was not confirmed until 1996[49,91]. Studies with earlier C1-esterase inhibitor formulations have shown a reduction in the frequency of attacks in patients with HAE[92]. In 1 study, more than 75% of patients were almost asymptomatic while receiving bolus intravenous (IV) injections of C1-esterase inhibitor 2 to 3 times weekly[93]. In a more recent study, 19 patients with HAE underwent weekly long-term therapy with C1-esterase inhibitor for an average of 9 years[94]. Disease severity was significantly improved by 1 or more injections of C1-esterase inhibitor per week; the percentage of severe attacks was 93.3% without and 3.8% with treatment[94]. To achieve this reduction in severity, patients were willing to accept 1 or more weekly IV injections. Although the majority of patients had fewer attacks during the last 12 months of the study, approximately one third reported more attacks. Signs of increasing disease activity (eg, multilocular edema) were reported in several patients, but no causal link was demonstrated in this observational study.

Efficacy data for the nanofiltered C1-esterase inhibitor approved for prophylactic therapy demonstrate an approximate 50% reduction in average normalized attack rates compared with placebo when administered over two, 12-week crossover periods (6.26 vs 12.73 attacks for C1-esterase inhibitor and placebo, respectively; difference 6.47 [95% confidence interval 4.21, 8.73]; *P *< 0.001)[43]. In the same study, prophylaxis with C1-esterase inhibitor demonstrated significantly lower severity of attacks (1.3 ± 0.85 vs 1.9 ± 0.36, *P *< 0.001) and significantly shorter duration of attacks (2.1 ± 1.13 vs 3.4 ± 1.39 days, *P *= 0.002) when compared with placebo, respectively[43]. Recent data from an open-label trial confirm this finding. Patients enrolled in the study had a decrease in the median number of HAE attacks from 3.0 per month to 0.2 per month, and 86% of patients had ≤ attacks per month[95].

The selection of a C1-esterase inhibitor over other prophylactic therapy options is a choice made as a result of consultation between physician and patient; the focus is on treatment failure or intolerance to other regimens or the inability to receive other prophylactic treatment options because of contraindications[37]. Similar to all drug therapies, C1-esterase inhibitor products also have safety concerns. In a clinical trial evaluating the use of 1 C1-esterase inhibitor (Berinert, CSL-Behring, King of Prussia, PA) in HAE attacks, the following adverse events were observed: headache, abdominal pain, nausea, muscle spasms, pain, diarrhea, and vomiting[42]. The safety results of a study evaluating the efficacy of nanofiltered human C1-esterase inhibitor (CINRYZE, ViroPharma Inc, Exton, PA) in prophylactic therapy show the most common adverse events to be sinusitis, rash, headache, and upper respiratory infection, irrespective of causality (Table 3)[46]. In the opinion of the investigators, the only adverse events that were possibly or definitely related to this C1-esterase inhibitor were pruritus and rash, lightheadedness, and fever[43]. In an open-label study, the adverse events most frequently related to nanofiltered C1-esterase inhibitor were headache (5.5%), nausea (4.1%), rash (2.7%), erythema (2.1%), and diarrhea (2.1%). The risk of experiencing a hypersensitivity reaction to the nanofiltered C1-esterase inhibitor is low[95]. An increased risk for thrombotic events was seen in preclinical animal studies and in neonates undergoing cardiovascular surgery from off-label use of extremely high doses of C1-esterase inhibitor therapy[96,97]. Studies conducted in humans show that a dose of up to 100 U/kg does not lead to thrombotic events[98,99]. Nevertheless, thrombotic events have been reported with C1-esterase inhibitors; patients who are at risk for thrombosis should be monitored while on C1-esterase inhibitor therapy. It is noteworthy to mention that discontinuation resulting from an adverse event associated with nanofiltered human C1-esterase inhibitor is rare; in a recently completed open-label study, no patient discontinued study therapy because of an adverse event[95].

**Table 3:** Adverse Events Observed in at Least 2 Subjects in a Randomized, Double-Blind, Placebo-Controlled Trial Evaluating Prophylactic Use of C1-Esterase Inhibitor Therapy Irrespective of Causality[46]

Adverse Event*	Number of Adverse Events	**Number of Subjects (N **= **24)**
Sinusitis	8	5
Rash	7	5
Headache	4	4
Upper respiratory tract infection	3	3
Viral upper respiratory tract infection	5	3
Bronchitis	2	2
Limb injury	2	2
Back pain	2	2
Pain in extremity	2	2
Pruritus	2	2

*There were no treatment-emergent serious adverse reactions observed in this trial.

Commercially available C1-esterase inhibitor products are derived from human blood and theoretically carry the risk of viral transmission (eg, parvovirus B19, hepatitis B, hepatitis C, and human immunodeficiency virus). Initial development of human plasma-derived C1-esterase inhibitor was complicated by the need for purification steps to reduce the transmission of viral diseases. Viral inactivation and removal steps in the production of C1-esterase inhibitor were introduced in the mid-1980s[85]. The approval of Berinert-P (CSL Behring GmbH, Marburg, Germany; C1-esterase inhibitor, human) in Europe in 1985 brought to market a formulation that used numerous steps to reduce viral transmission, including careful donor screening and viral contaminant elimination and inactivation through adsorption, precipitation, and pasteurization steps[92,100]. More recently, manufacturing of another C1-esterase inhibitor formulation has incorporated a nanofiltration step through 2 serial 15-nm filters to reduce transmission of enveloped and nonenveloped viruses and possibly prions[43,101]. It is important to note that, as a result of these purification measures, no cases of virus transmission have been attributed to the purified preparations of C1-esterase inhibitor currently available[42,46,87,95,100,102].

## Clinical Experience Discussion

The manifestations and severity of HAE are highly variable and unpredictable, which necessitates individualized therapeutic approaches. Worldwide, a variety of treatment options are used to manage the disease. On-demand therapy for attacks with C1-esterase inhibitor is sometimes used as a patient's sole method of treatment or it may be used to treat breakthrough attacks in patients who are taking long-term prophylaxis[103]. Home therapy refers to treatment given by the patient (self-administration) or a trained caregiver outside of a healthcare facility. On-demand home therapy with C1-esterase inhibitor has proven successful in selected patients and offers patients the possibility of earlier treatment and better symptom control[103-106]; however, this use is not currently approved in the United States. For prophylaxis, oral attenuated androgens or IV C1-esterase inhibitor are at the forefront. Home therapy is only approved with the nanofiltered C1-esterase inhibitor that is approved for routine prophylaxis against angioedema attacks in adolescents and adults in the United States[46].

**Figure 4:**
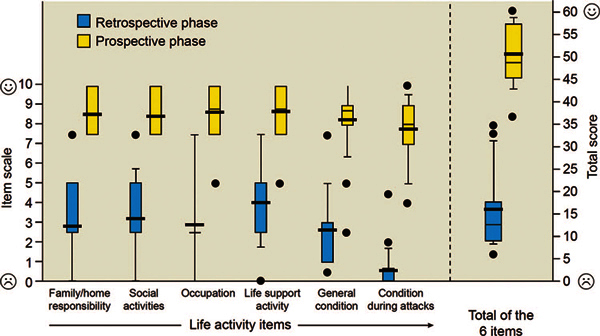
**Quality-of-life improvements: life activity items and total score (standardized items on an 11point scale)**. All item values achieved during C1-esterase inhibitor therapy improved significantly compared with danazol prophylaxis (Wilcoxon signed rank test; *P *< 0.001) resulting in a significantly improved total score for patients receiving C1-esterase inhibitor therapy. Bold bars in the middle of the box represent mean values and normal bars stand for median values. Prospective phase = C1-esterase inhibitor therapy; retrospective phase = danazol therapy (reproduced from Kreuz et al,[89] with permission).

Undoubtedly, clinicians must take into consideration the benefits and risks of an agent when evaluating treatment options for long-term prophylaxis of HAE. This should include a careful assessment of treatment impact on patient quality of life. One study conducted by Kreuz and colleagues[89] demonstrated that patients with severe HAE who discontinued long-term prophylaxis with danazol because of lack of efficacy, intolerability, or severe side effects had a significant improvement in quality-of-life scores after receiving C1-esterase-inhibitor therapy (Figure 4)[89]. Although these results should not be generalized to the larger HAE population because the enrolled patients were refractory to danazol therapy, they emphasize the negative impact that lack of efficacy or adverse effects can have on patients. Here we discuss our clinical experience with attenuated androgens and C1-esterase inhibitor and their appropriateness for long-term prophylactic use on a case-by-case basis.

## Case 1: Long-Term Prophylaxis With C1-Esterase Inhibitor

A 10-year-old girl presented to the emergency department (ED; case provided by Dr William Lumry, USA) complaining of inability to swallow. Her symptoms began 2 hours before arrival. She reported awakening with a feeling of fullness in her throat that progressed to scratchiness on her tongue. Her mother, seeing nothing unusual in her daughter's mouth, assumed she was having postnasal drainage from seasonal allergies and gave her diphenhydramine. One hour later the girl was spitting saliva in a cup and complaining of a tight throat and inability to swallow. The mother took her to the ED. Initial questioning revealed there was no history of similar events and the patient had neither insect-bite exposure nor new medications or foods in the prior 24 hours. She had a history of seasonal allergic rhinitis with typical symptoms, but no history of food or drug allergies. The patient took antihistamines as needed for seasonal allergies and a multivitamin daily. She had been in the ED 2 months previously because of intractable vomiting that began 6 hours after being hit in the stomach by a soccer ball.

Vital signs consisted of a blood pressure of 100/66 mm Hg, heart rate 100, respirations 20, and 90% oxygen saturation on room air; she was afebrile. Physical examination was normal with a few exceptions: her voice was hoarse, her head was tilted forward, the posterior pharynx and uvula were edematous, and inspiratory stridor was heard over the trachea. No facial, tongue, lip, or neck swelling or cervical adenopathy or tenderness were noted.

On arrival, the patient received oxygen supplementation, IV diphenhydramine HCl 50 mg, IV methylprednisolone 60 mg, and epinephrine (1:1000) 0.3 mL given intramuscularly. The patient's symptoms did not change with these maneuvers.

Laboratory evaluation included a normal complete blood count and chemistry panel. Chest x-ray was normal, however, soft-tissue lateral neck film revealed straightening of the cervical spine with edema of the epiglottis and upper airway.

The mother subsequently revealed a family history of attacks of throat swelling, episodic hand and foot swelling, and abdominal pain. Both the mother and a maternal aunt had been diagnosed with HAE in their teenage years. The patient had recently been found to have C1-esterase inhibitor deficiency consistent with a diagnosis of HAE, although she had not had any apparent swelling attacks.

After the family history of HAE was revealed, the mother's physician was called. The child had been diagnosed with type 1 HAE and enrolled in a clinical study for treatment of attacks of HAE. Her laboratory results on entry to the study were C4 = 4 mg/dL, C1-esterase inhibitor = 8 mg/dL, and functional C1-esterase inhibitor 22%. Open-label study drug was provided by the physician. The patient was given 1000 units of nanofiltered C1-esterase inhibitor by IV infusion over 10 minutes. Within 30 minutes, the patient was able to swallow.

In light of this potentially life-threatening laryngeal HAE attack, the child's physician prescribed routine prophylaxis with C1-esterase inhibitor concentrate. Currently, the patient receives infusions of 1000 units of nanofiltered C1-esterase inhibitor every 3 to 4 days. She has experienced no severe HAE swelling attacks since beginning prophylactic therapy 1 year ago.

Although diagnosing HAE may be difficult because of variability in clinical presentation, this case illustrates the importance of having a high level of suspicion for HAE in patients presenting with characteristic symptoms, especially in the presence of a positive family history[25]. It is recommended that symptom-free children with a positive family history have an initial screening (including complement tests) at the age of 6 months and again at 1 year of age[64].

Patients experiencing laryngeal edema often complain of tightness of the throat, inability to clear saliva, and dysphagia. Patients may also have edema of the soft palate or tongue, making swallowing and breathing difficult[37]. Because laryngeal edema can be fatal, prompt treatment is essential[33]. As seen with this patient, misdiagnosis of HAE symptoms is common. Standard therapies for allergy-mediated angioedema (eg, epinephrine, corticosteroids, and antihistamines) are not effective in treating HAE symptoms because HAE manifestations are mediated by bradykinin whereas angioedema caused by allergic reactions is mediated by histamine and other mediators[9]. In this case, the patient's laryngeal edema did not respond to standard therapies for an allergic reaction. This case also highlights the need for patient and caregiver education about HAE symptoms and emergency care procedures. Although the mother was diagnosed with HAE and was aware her child had a C1-esterase inhibitor deficiency, there were delays in obtaining medical attention and in providing family and patient history to medical personnel.

Long-term prophylaxis is indicated for patients who experience frequent or severe attacks, laryngeal attacks, have significant anxiety or poor quality of life as a result of HAE, or have limited access to emergency medical care[37]. The fact that this patient has not experienced severe HAE swelling attacks since beginning prophylactic therapy underscores the value of this therapy in the management of HAE. Nevertheless, because the clinical course of HAE demonstrates vast variability over the lifetime of an individual patient, the need for prophylactic therapy should be reassessed at regular intervals.

## Case 2: Long-Term Prophylaxis With Attenuated Androgen

A 35-year-old female patient experienced periodic episodes of peripheral edema of the hands or feet with occasional edema around the waist beginning at 7 years of age (case provided by Dr Teresa Caballero, Spain). Most episodes were accompanied by abdominal symptoms, including abdominal bloating, nausea, and vomiting. Occasionally the attacks were preceded by a skin rash consistent with serpiginous erythematous macules with a clear center. Family history was negative for angioedema; she had a personal history of seasonal rhinoconjunctivitis. The patient was initially evaluated for a rheumatic disorder and for drug allergy.

In 1996 at the age of 21, she was diagnosed with HAE caused by C1-esterase inhibitor deficiency. Danazol 200 mg every 8 hours was prescribed and symptoms were well controlled. The dose of danazol was progressively reduced to 200 mg 3 days a week. In 2000, she was referred to a comprehensive HAE center for evaluation. The patient did not experience any secondary effects related to attenuated androgen therapy (ie, she did not experience amenorrhea, weight gain, hypertension, dyslipidemia, voice change, or hirsutism). Evaluation revealed that precipitant factors included menses, upper respiratory tract infections, and oral contraceptives containing estrogens.

Hemogram, erythrocyte sedimentation rate (ESR), blood biochemistry (cholesterol, triglycerides, AST, ALT, others), and urinalysis were all within normal limits. Blood serologies were negative for human immunodeficiency virus (HIV), hepatitis C virus (HCV), and parvovirus. Serology testing for hepatitis B virus (HBV) was positive for anti-HBs antibodies with negative anticore antibodies and negative HbS antigen, which is concordant with HBV postimmunization.

Results of complement studies were C4 = 2.68 mg/dL (normal 14 to 47 mg/dL), C1-esterase inhibitor <4.8 mg/dL (normal 14 to 40 mg/dL), and functional C1-esterase inhibitor 24% (normal 70 to 120%); values were normal for C3 and C1q. Complement analysis was normal in her parents and siblings. Genetic studies were performed in the patient and her parents, and a mutation was confirmed in the patient, but was not present in either of her parents.

The patient was diagnosed with HAE caused by C1-esterase inhibitor deficiency (spontaneous mutation/de novo mutation). Although her HAE symptoms had been well controlled with danazol and she did not experience adverse effects from the drug, the prophylaxis treatment was changed to stanozolol 2 mg 3 days a week based on our positive experience with this drug and the evidence that it is associated with fewer adverse effects[70]. Plasma-derived human C1-esterase inhibitor (Berinert, CSL-Behring, Marburg, Germany) was recommended for attacks and medication was given for home storage. The patient was advised to increase the dose of stanozolol in the event of an upper respiratory tract infection or other known precipitant factors. Plasma-derived human C1-esterase inhibitor was also recommended for short-term prophylaxis before medical or surgical interventions.

The patient has been followed every 6 months on an outpatient basis for the past 10 years. Her disease has been well controlled, with 0 to 2 mild edema attacks per year. Blood and urine analyses have been conducted every 6 months and an abdominal ultrasound has been performed annually. Occasional transient mild hypercholesterolemia (222 mg/dL) (normal <200 mg/dL) and occasional transitory elevation of hepatic enzymes (ALT 48 UI/L) (normal <30 UI/L) have been detected. Other laboratory investigations have been within normal limits. No changes have been detected on abdominal ultrasound.

This patient is a good example of the variability of clinical expression of HAE disease and the possibility of controlling disease with low doses of attenuated androgens in some patients. Although the patient had exhibited obvious symptoms since the age of 7 years, she was not diagnosed until 21 years of age. Although many patients with HAE have delayed diagnoses,[107] the elapsed time between initiation of symptoms and diagnosis in this patient was 14 years, which may be attributed to the absence of family history of HAE. Diagnosis may be delayed in patients with de novo mutation, which may be present in up to 25% of HAE patients, because of the lack of family history[108].

Until recently, attenuated androgen therapy was the only approved prophylaxis treatment option for HAE in the United States[58]. This patient's disease has been controlled with low doses of attenuated androgen (first danazol and later stanozolol) for more than 10 years, with a maintenance dose as low as stanozolol 2 mg 3 days per week. Although attenuated androgens are associated with significant adverse effects,[59] this patient has tolerated treatment well. In her case, the only reported adverse effects that may be associated with stanozolol were mild hypercholesterolemia and mild elevation of hepatic enzymes, both of which spontaneously normalized. Attenuated androgens also provide the convenience of oral dosing.

This case also exemplifies the need for vigilance and thorough monitoring while on androgen therapy as outlined by the International Consensus Algorithm for the Diagnosis, Therapy, and Management of Hereditary Angioedema[25]. Patients receiving attenuated androgen therapy should be evaluated every 6 months for changes in liver function, lipid profile, complete blood count, and urinalysis. Periodic ultra-sounds of the liver and spleen are also recommended.

## Case 3: Intermittent Prophylaxis

The patient is a 33-year-old female (case provided by Dr Henriette Farkas, Hungary). At the age of 4 years the patient developed extremity edema after minor mechanical trauma that resolved spontaneously within 2 days. Subsequently, once or twice a year the patient experienced edematous episodes of several days' duration involving the upper or lower extremities. Edema always resolved spontaneously and its cause could not be identified. Appendectomy was performed at the age of 7 years, and intraoperative findings included free peritoneal fluid and edematous intestines. At 10 years of age, the patient experienced facial edema after a tonsillectomy. Edema was treated with antihistamines and glucocorticoids and resolved slowly over 3 days. This event raised the suspicion of HAE. Clinical findings, a positive family history (the patient's mother died of suffocation from laryngeal edema at the age of 32), and the results of complement testing (C4: 0.02 g/L [normal 0.15 to 0.55 g/L]; C1-esterase inhibitor antigenic level: 0.03 g/L [normal 0.15 to 0.3 g/L]; C1-esterase inhibitor functional activity: 18% [normal 70 to 100%]; C1q: 118 mg/L [normal 60 to 180 mg/L]) confirmed the diagnosis of type I HAE. The patient was enrolled in a follow-up program with 2 prescheduled control visits each year.

At the age of 13, the frequency and severity of edematous episodes increased. Attacks recurred on 2 or 3 occasions per month and an abdominal attack occurred. The increased frequency of attacks was attributed to the physiological hormonal changes of puberty. A trial of prophylactic therapy with tranexamic acid was ineffective, and therefore, danazol 200 mg daily was introduced. This relieved symptoms considerably, with the number of attacks decreasing to as low as 2 to 3 extremity attacks and 1 abdominal attack per year. The patient tolerated pharmacotherapy well. Adverse effects included transient episodes of irregular menses and headache, but these were not severe enough to warrant discontinuation of treatment. The dose of danazol was reduced to 100 mg every other day.

An unplanned pregnancy occurred at the age of 23 and danazol was discontinued. This was followed by a dramatic increase in attack frequency; edematous episodes recurred 2 to 3 times per week and 80% of the attacks were of the combined type (severe abdominal attack with extremity edema). Laryngeal edema developed on a single occasion. C1-esterase inhibitor administered during attacks was effective; it mitigated symptoms within 15 to 30 minutes. Thus, intermittent C1-esterase inhibitor prophylaxis was introduced in the eighth week of gestation. Administration of 500 IU C1-esterase inhibitor twice a week achieved near-complete elimination of symptoms, with only 1 episode of mild extremity edema that occurred after mechanical trauma. The patient did not experience any adverse reactions to therapy. After the 12th week of pregnancy, the dose of C1-esterase inhibitor was reduced to 500 IU weekly and then, as the patient had become symptom-free, C1-esterase inhibitor prophylaxis was discontinued at week 15. The patient gave birth by vaginal delivery to a healthy neonate during the 38th week of gestation; the child has not inherited HAE. Although no edema developed during delivery, C1-esterase inhibitor was kept at hand. Long-term prophylaxis was not necessary during the 2-month lactation period. Several instances of mild subcutaneous edema and a single abdominal attack occurred; the latter responded to acute treatment with 500 IU of C1-esterase inhibitor. The patient remained symptom-free for 2 years after the lactation period. Subsequently, however, increasing attack frequency and severity (with no identifiable cause) required the reintroduction of danazol prophylaxis. Currently, the patient is on danazol 100 mg every other day.

This case illustrates that long-term prophylaxis of HAE does not necessarily mean uninterrupted drug therapy over a lifetime. During regularly scheduled check-ups, the therapeutic regimen can be modified: drugs may be discontinued, others may be introduced, and dosages may be adjusted. In this case, tranexamic acid was ineffective, and therefore, switching to danazol was necessary. Titration to the lowest effective dose (in this patient from the daily 200-mg initial dose to 100 mg every other day) is recommended to avoid or at least mitigate adverse effects[2].

Precipitating factors such as stress, trauma, infection, menstruation, and pregnancy have been reported to contribute to the frequency of HAE attacks[3,4,9,21,23]. Short-term prophylactic treatment, also referred to as procedural prophylaxis, is advised for patients with a planned exposure to a situation that may trigger an attack, such as dental work or invasive medical procedures[2,25]. The care of pregnant women, however, is complicated by the potential adverse effects of attenuated androgens on the developing fetus[2]. In this patient, danazol was discontinued when she became pregnant and attack frequency increased dramatically. Recent consensus guidelines proposed that C1-esterase inhibitor is the safest prophylactic agent during pregnancy[25]. In this patient, intermittent prophylaxis with C1-esterase inhibitor during pregnancy proved a safe and effective therapy. Although intermittent prophylaxis of HAE is not mentioned in relevant clinical guidelines, it may be a safe and effective treatment modality in children[64] and in cases in which the identifiable cause of increased attack frequency and severity (such as pregnancy in our case) cannot be eliminated.

## Case 4: Optimizing Therapy for Hae

The patient is a 45-year-old male welding instructor at a technical college (case provided by Dr Douglas Johnston, USA). His family history is positive for HAE; his father died of a laryngeal HAE attack and his sister, nephew, and son have HAE. The patient presented with recurrent intermittent bouts of abdominal pain. These episodes, which began at 8 years of age, are described as crampy discomfort that lasts for 3 to 4 days despite medications and resolve on their own. The patient was diagnosed with HAE at 16 years of age after being hospitalized for a severe abdominal episode. Initial treatment was with methyltestosterone, but this was changed to stanozolol for greater efficacy. Stanozolol was switched to danazol 15 years ago; however, the patient began experiencing more frequent swelling episodes and approximately 4 severe abdominal episodes a year while receiving danazol 200 mg daily. Triggers for the attacks include upper respiratory infections, alcohol, amoxicillin, pizza, and yeast rolls. The patient continued to take danazol 200 mg daily, but experienced recurrent swelling of the hands and feet approximately 8 to 10 times per year, with each episode lasting between 3 and 5 days. These episodes were often mild and did not require medical attention. Laboratory values were C1-esterase inhibitor of 5 mg/dL, functional C1-esterase inhibitor at 1%, C4 of 5 mg/dL, and normal AST and ALT. An ultrasound of the right upper quadrant was notable for 3 small hepatic hemangiomata.

Long-term prophylactic therapy was changed to stanozolol 2 mg daily, which led to less frequent abdominal attacks. The patient continued to have hand swelling, and although the frequency of attacks was decreased, the patient missed work as a result of the attacks. While taking stanozolol, AST and ALT increased to 48 and 71 IU/L, respectively, blood pressure and low-density lipoprotein increased, and high-density lipoprotein decreased. Therapy was changed to C1-esterase inhibitor 1000 units twice a week. During the past 6 months, the patient has had 2 episodes of abdominal swelling, which were less intense than his previous episodes. One was precipitated by trauma and the other was attributed to eating several slices of pizza. The patient reports that when he has forgotten to administer a dose of C1-esterase inhibitor, he suffers from hand swelling, which functions as a reminder to take his medication. The swelling often resolves within 1 hour after administration of the dose. His AST and ALT are normal (25 and 29 IU/L, respectively), and high-density lipoprotein has increased from 31 to 39 mg/dL.

This case highlights the need for optimizing therapy for HAE. Treatment of HAE should be individualized and based on an assessment of the severity, frequency, and nature of the attacks. This, in combination with the patient's circumstances, should guide the development of an appropriate treatment plan. This patient experienced frequent attacks that had a negative impact on his quality of life, making him a candidate for long-term prophylaxis[37]. Despite prophylactic therapy with danazol, he continued to have frequent attacks. After therapy was changed to stanozolol the frequency of the attacks decreased; however, his attacks continued to result in work absences. Furthermore, his laboratory tests indicated lipid abnormalities and increased liver enzymes, which are adverse effects associated with attenuated androgen therapy[59,62,75]. Therapy was changed to C1-esterase inhibitor in an attempt to provide a better clinical outcome and to avoid the deleterious effects of attenuated androgen therapy. The number and severity of his attacks have decreased with C1-esterase inhibitor therapy and his laboratory values have improved. This case underscores the importance of the physician-patient relationship in the management of HAE to optimize pharmacotherapy and to improve education of the patient. Although the patient recognizes certain triggers for his attacks, he should be reminded to avoid stimuli that may precipitate attacks to substantially improve disease control[2]. Furthermore, compliance with therapy should be emphasized because this will likely lessen the number of attacks suffered by the patient.

## Summary: Implications for the Selection of Prophylactic Therapy for Hae

HAE is a serious disease that may cause life-threatening and disabling symptoms, which can have a dramatic impact on quality of life. Because the manifestations and severity of HAE are highly variable and unpredictable, patients need individualized care to reduce the burden of HAE on daily life. Furthermore, differences exist between European and US interventions for HAE primarily because of differences in product availability. On-demand therapy for attacks with C1-esterase inhibitor is sometimes used as a patient's sole method of treatment or it may be used to treat breakthrough attacks in patients who are taking long-term prophylaxis. Routine long-term prophylaxis with either attenuated androgens or C1-esterase inhibitor has been shown to reduce the frequency and severity of HAE attacks. Therapy with attenuated androgens, a mainstay of treatment in the past, has been marked by concern about potential adverse effects. C1-esterase inhibitor works directly on the complement and contact plasma cascades to reduce bradykinin release, which is the primary pathologic mechanism in HAE. As our patient cases illustrate, different approaches to long-term prophylactic therapy can successfully provide HAE management that is tailored to meet the needs of the individual patient.
